# Civilian Surgeons in Time of War

**Published:** 1901-06-29

**Authors:** 


### HOSPITAL CLINICS AND MEDICAL PROGRESS.

### CIVILIAN SURGEONS IN TIME OF WAR.

COMMENTING on the suggestions contained in the report of the Hospital Commission Mr. Watson Cheyne[1] deals with the question how to utilise the civilian auxiliaries which in any great war must of necessity be called upon to assist or to supplement the Royal Army Medical Corps. Whatever may be done to increase the numerical efficiency of the Army Medical Department one cannot conceive of any Government attempting to maintain in time of peace a medical staff sufficiently large to cope unassisted with the strain which would be thrown upon it by any great war, and Mr. Watson Cheyne very properly insists that the connection between the military and civilian element should not be a merely temporary arrangement, made on the spur of the moment to get over an immediate difficulty, but should form part and parcel of the permanent provision for the medical service of the army. Bringing to bear upon the problem the experience gained in the present war, and what he has himself seen as to the management of the hospitals in South Africa, he urges that a very marked distinction should be drawn between the base hospitals and those at the front, and that while the medical service in the field should be exclusively military, that at the base should be as entirely civilian as is compatible with maintaining its connection with the Department. He points out with great force that during the present war a not inconsiderable number of men, both on the military and the civilian side, have been employed in doing work for which they were by no means specially fitted by their training, and for which in some cases they were absolutely unfitted. In the present campaign in South Africa the civil surgeons were more or less mixed up with the army surgeons. Both base and field hospitals contained both military and civil surgeons, and in times of stress civil surgeons had to be sent in charge of regiments or otherwise employed in the field in the place of the trained men of the Royal Army Medical Corps. Civil surgeons were, in fact, temporarily incorporated with the R.A.M.C., and were treated as belonging to that corps. That they did their work well has been universally acknowledged. But there were difficulties in their position. They had no experience of military matters and of the multifarious duties which a surgeon at the front may at any time be called upon to perform. Thus it might and did happen that the civil surgeon had to do things of which he had no knowledge. He might, for example, be left in charge and have to supply his unit with rations, equipment, transport, &c., without knowing how to go about it, which was a risky experiment. On the other hand a considerable number of army medical officers and men who, by their training, were thoroughly capable of doing the work at the front, were retained at the base hospitals (and this applies especially to the orderlies) doing work for which the civilian auxiliaries, both doctors and nurses, were admirably fitted. So far as the nursing was concerned, Mr. Watson Cheyne holds that if at Capetown, Bloemfontein, Johannesburg, &c., the large base hospitals could have been put in the hands of civilians with a few army men attached to act as connecting links with the army, and if they had been nursed by women as are the civil hospitals in London, a large number of efficient men would have been set free for work at the front and there would have been but little necessity to recruit the orderly staff from partially or entirely untrained men. To put the matter shortly he holds that the place of the men of the Royal Army Medical Corps is at the front and the place for the civilian is at the base, and that matters ought to be so organised that this division of labour should be maintained automatically. "I do not think," he says, "that the mixture of the two both at the base and at the front works well." He seems to us merely to express in words what has again and again happened in fact, when he says that to put a good civil surgeon at a base hospital under a less efficient Royal Army Medical Corps man (and the Royal Army Medical officers are selected by seniority rather than by professional ability) is to lead to friction or to breed contempt of his superior officer in the mind of the civil surgeon; while, on the other hand, to put a civilian at the front where he has to do work connected with obtaining transport, equipment, rations, &c., with the details of which he is not acquainted, is to irritate the Army Medical officer in charge. Mr. Watson Cheyne advises then that the army men and the civilians should not be mixed; that the base hospitals should be run on civil lines with surgeons and physicians of high standing in principal charge, with a good staff of assistants and a plentiful supply of women nurses; and that the link with the army should be kept up by an army medical officer being attached to each hospital. If this were done he thinks that there would be no necessity for the appointment of consulting surgeons, for the civilian surgeons and physicians placed at the head of these great hospitals would be men of the same standing as the consulting surgeons, and could be made available for consultation if required. But—and this seems a very important point—all these arrangements should be made part of the permanent organisation of the army medical department, and even in times of peace the senior civilians embraced in this organisation should be identified more or less with the army medical department, so that when sent out to take charge there should be no feeling that they were supplanting the regular officers, or any implication that the *personnel* of the R.A.M.C. were incapable of carrying on their work. There can, we think, be no doubt that an organisation somewhat on the lines suggested by Mr. Watson Cheyne will have to be adopted if the immense mass of professional skill and experience always existing in the civilian branches of the profession are to be rendered available for military purposes.

[1] Brit. Med. Jour., June 22.

